# Supplementary material for: Impact of bacterial culture medium on composition and characteristics of Burkholderia pseudomallei extracellular polymeric substances
Source: PeerJ. 2025 Dec 16;13:e20488. doi: 10.7717/peerj.20488 (PMC12716132; doi:10.7717/peerj.20488)
Supplement: Supplemental Information 2 [file peerj-13-20488-s002.docx]

**Supplementary Table S2** FTIR absorption bands and corresponding functional groups identified of EPS extracted from *B. pseudomallei* biofilms grown in BHI, LB and MVBM media for 6 days.

| **Wave Number (cm^-1^)** | **Assignment** | **Band Position (cm^-1^)** | | |
| --- | --- | --- | --- | --- |
|  |  | **Culture media** | | |
|  |  | **BHI** | **LB** | **MVBM** |
| 3400–3200 | O–H stretching of hydroxyl groups and N–H stretching | 3400.53  3276.18 | 3272.48 | 3261.18 |
| 3000–2800 | C–H stretching of –CH3 and>CH2 functional groups dominated by fatty acid chains | 2931.00 |  | 2924.36 |
| 1700–1600 | C=O stretching | 1637.40 | 1636.64 | 1599.41 |
| 1500–400 | Fingerprint region | 1364.50  1242.53  1140.81  1046.84  939.76  854.45  833.84  772.95  719.14 | 1368.92  1318.62  1050.79  929.40  866.53  834.24  772.00 | 1368.73  1050.39  985.84  933.58  854.39  834.35 |

FTIR Wave Number (cm^-1^):

**-** 120–1260, 1080–1090, 1700–1600 (DNA: indicate the presence of phosphate groups and heterocyclic bases)

- 3400, 2930, 1150–1000, 890 (Polysaccharides: O–H, C–H, C–O–C, glycosidic bonds)

- 1650, 1540, 1230 (Proteins: Amide I, II, III (peptide linkages))
